# Problem Management Plus for improving mental health among earthquake-affected individuals in Türkiye: pilot randomised controlled trial

**DOI:** 10.1192/bjo.2026.12016

**Published:** 2026-06-15

**Authors:** Ayşenur Coşkun Toker, Gülşah Kurt, Gökhan Malkoç, Ceren Acarturk

**Affiliations:** Department of Psychology, Koç University, Istanbul, Türkiye; Nutrition, Exercise & Social Equity (NExuS) Research Group, Discipline of Psychiatry and Mental Health, The University of New South Wales Sydney, Australia; Department of Psychology, Istanbul Medipol University, Türkiye

**Keywords:** Earthquake survivors, mental health, randomised controlled trial (RCT), Problem Management Plus (PM+), feasibility

## Abstract

**Background:**

Earthquakes present significant threats to community mental health, yet post-disaster resource limitations and high demand restrict access to care. The 2023 Türkiye–Syria earthquakes affected millions, increasing psychological problems and highlighting the urgent need for scalable interventions.

**Aims:**

This study assessed (a) the feasibility of procedures prior to a fully powered randomised controlled trial (RCT) and (b) the potential effectiveness of Problem Management Plus (PM+), a scalable psychological intervention, in reducing common mental health problems among Turkish earthquake survivors.

**Method:**

A two-arm, single-blind pilot RCT was conducted with survivors residing in temporary housing in Türkiye. Participants were randomly allocated (1:1) to PM+ (*n* = 38) or enhanced care-as-usual (E-CAU; *n* = 38) and assessed at baseline, 1 week post intervention and at the 1-month follow-up. The primary outcome was depression (Patient Health Questionnaire, PHQ-9); secondary outcomes were self-identified problems (Psychological Outcome Profiles, PSYCHLOPS), functional impairment (World Health Organization Disability Assessment Schedule, WHODAS 2.0), psychological distress (Kessler-10 Psychological Distress Scale, K-10), anxiety (Generalised Anxiety Disorder Scale, GAD-7) and post-traumatic stress (Post-traumatic Stress Disorder Checklist, PCL-5). The trial was prospectively registered (NCT06026306).

**Results:**

Recruitment, retention (74%) and treatment adherence (79%) supported feasibility. At post-assessment, PM+ showed greater reductions than E-CAU across all outcomes: PHQ-9 (adjusted mean difference −5.91, 95% CI –8.23 to –3.58), PSYCHLOPS (−6.94, –8.88 to –5.01), WHODAS 2.0 (−8.77, –12.50 to –5.03), K-10 (−9.66, –13.35 to –5.97), GAD-7 (−4.31, –6.39 to –2.24) and PCL-5 (−18.72, –25.97 to –11.47). At follow-up, improvements remained significant for psychological distress and self-identified problems.

**Conclusions:**

Findings provide preliminary evidence that PM+ is a feasible and potentially effective intervention for alleviating common mental health problems among Turkish earthquake survivors.

## Mental health consequences of earthquakes

Earthquakes are among the most devastating natural disasters, striking without warning and leaving behind not only physical destruction but also profound psychological challenges. Earthquake survivors, in particular, experience higher rates of trauma-related psychopathology over time compared with those affected by other types of disasters.^1^ Meta-analytical evidence indicates considerable heterogeneity in post-earthquake mental health outcomes depending on timing, exposure severity and contextual factors. Post-traumatic stress disorder (PTSD) prevalence ranges from 1.20 to 82.64% across studies.^2^ In specific high-impact earthquake contexts, such as the 2010 Haiti earthquake, prevalence estimates for severe depression and anxiety have been reported as 32.16 and 20.49%, respectively.^3^ On 6 February 2023, 2 major consecutive earthquakes (Mw 7.7 and 7.6) hit the Türkiye–Syria region, causing severe destruction across 11 provinces. More than 50 000 lives were lost, with over 15 million people – around 16% of the population – affected,^4^ making this the worst natural disaster in the WHO European Region in a century.^5^ Recent studies reported high levels of mental health problems among affected populations in Türkiye. PTSD prevalence rates ranged from 17.4 to 54.1% across different samples.^6–8^ Depression, anxiety and psychological distress were also highly prevalent, with some studies reporting rates of 61.2% for depression, 79.4% for anxiety and 58.4 for distress.^7^ Longitudinal studies indicate that these problems can persist for years or even decades if untreated,^9^ with depression in particular showing little improvement over time.^10^ They lead to significant functional impairment such as unemployment, social withdrawal, substance misuse and increased household tension,^11^ often sustained by ongoing post-disaster stressors, including displacement, poor housing conditions, disrupted social networks and difficulties accessing basic needs.^9,10^

## Challenges in post-disaster mental health services

Taken together, these findings highlight the need for addressing mental health challenges faced by earthquake survivors and establishing appropriate response systems.^12^ Post-disaster mental health and psychosocial support (MHPSS) typically includes a wide array of activities, from basic psychosocial support to specialised clinical care,^13^ yet they are often fragmented, short-term and focused on acute needs. Sustained implementation is limited by workforce shortages, unstable funding and difficulties integrating these services into national systems.^14,15^ These challenges are particularly pronounced in low-resource settings, where pre-existing gaps in infrastructure and coordination further hinder mental health care during emergencies.^16,17^ In Türkiye, most disaster-affected areas are socioeconomically disadvantaged and experienced severe infrastructure collapse after the earthquakes. In these areas, the delivery of mental health services was further hampered by heavy reliance on non-governmental organisations (NGOs), shortages of trained staff and funding, and the absence of unified protocols.^18^

## Problem Management Plus (PM+)

To improve access to evidence-based psychological support in resource-limited crisis settings, the World Health Organization (WHO) developed brief, scalable transdiagnostic psychological interventions within its Mental Health Gap Action Programme.^19^ Among these, Problem Management Plus (PM+) is a low-intensity intervention designed to address a range of emotional and practical problems associated with adversity, and is positioned within the ‘focused, non-specialised supports’ level of the WHO Inter-Agency Standing Committee (IASC) MHPSS pyramid.^20^ PM+ consists of five 90 min sessions and can be delivered by trained laypersons in individual or group formats. It is based on evidence-based strategies, including stress management, problem solving, behavioural activation and increasing social support. Several RCTs have shown the effectiveness of PM+. It was found effective in reducing the symptoms of depression, anxiety and PTSD in conflict- and natural disaster-affected regions of Pakistan^21^ and psychological distress among females affected by gender-based violence in Kenya.^22^ Group PM+ (gPM+) has also shown benefits across diverse post-conflict and disaster-affected populations.^23–26^ A recent meta-analysis found that PM+ was effective in reducing mental health problems, with a small to moderate effect (mean difference −0.45, 95% CI −0.56 to −0.34) and benefits sustained in the long run.^27^ Considering that approximately 16% of Türkiye’s population was affected by the earthquakes, addressing the mental health needs poses a significant challenge, and recovery requires long-term support. Given the fragmented service structures and the ongoing psychosocial burden, a brief, structured, transdiagnostic and evidence-based intervention such as PM+ offers a feasible and scalable strategy. Its approach directly targets the stressors and functional impairments frequently reported by earthquake survivors. Furthermore, since collaboration with local organisations is considered essential for sustainability and uptake of interventions in humanitarian settings,^28^ partnering with a local implementation network may support feasibility and potential scale-up.

## The study rationale

The aim of this pilot RCT was twofold: (a) to assess the feasibility of trial procedures – including randomisation, recruitment, data collection and retention – prior to conducting a fully powered RCT and (b) to provide preliminary evidence on the potential effectiveness of PM+ for reducing common mental health problems among earthquake survivors residing in temporary housing in Türkiye. To our knowledge, this is the first pilot RCT to investigate PM+ in the Turkish population. Evaluating the intervention in this context may provide preliminary evidence to inform its potential integration into MHPSS systems and guide future implementation in disaster settings. We hypothesised that PM+ participants would show greater improvement in depression (primary outcome) at 1 week post treatment, and greater reduction in self-identified problems, psychological functioning, psychological distress, anxiety and post-traumatic stress symptoms at both 1 week post treatment and the 1-month follow-up assessment.

## Method

### Study design

This study was a two-arm, single-blind pilot RCT in which PM+ was compared with enhanced care-as-usual (E-CAU). The authors assert that all procedures contributing to this work comply with the ethical standards of the relevant national and institutional committees on human experimentation and with the Helsinki Declaration of 1975, as revised in 2013. All procedures involving human subjects/patients were approved by the Research Ethics Committee of Koç University (Protocol ID: 2023.271.IRB3.125) and prospectively registered (ClinicalTrials.gov Identifier: NCT06026306). This study is reported based on Consolidated Standards of Reporting Trials (CONSORT) guidelines (Supplementary Material 1, Table S1). There was no patient or public involvement in the design, conduct or reporting of this study.

### Setting and study population

The study was conducted in collaboration with the Earthquake Psychosocial Solidarity Network (Deprem Psikososyal Dayanışma Ağı, DEPSDA) of the Turkish Psychological Association, a local NGO initiative aimed at providing psychosocial support in earthquake-affected regions. The study was implemented in four container cities, which are temporary container-based housing settlements, in different earthquake regions (i.e. Adıyaman, Kahramanmaraş, Malatya, Osmaniye). Participants were recruited by informing residents of these settlements through the psychosocial staff of DEPSDA, who announced the study during psychosocial support activities and approached residents interested in receiving support. Participants were recruited from all four locations with a similar distribution across sites (Adıyaman *n* = 19, Kahramanmaraş *n* = 17, Malatya *n* = 20, Osmaniye *n* = 20).

**Fig. 1:**
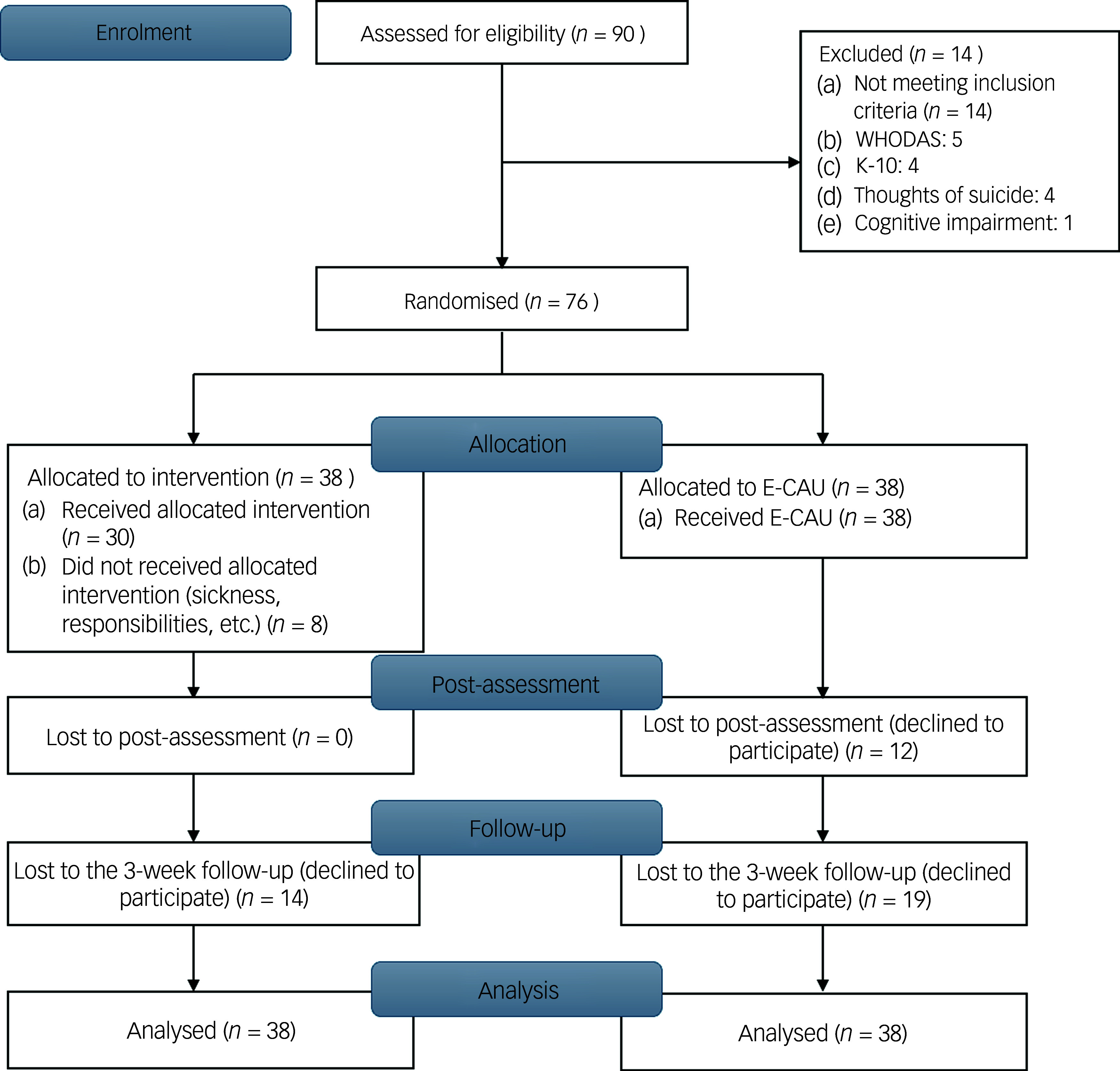
Fig. 1 long description. Consort flowchart. WHODAS, World Health Organization Disability Assessment Schedule; K-10, Kessler-10 Psychological Distress Scale; E-CAU, enhanced care-as-usual.

Earthquake survivors who gave written informed consent to participate were screened to assess whether they are eligible to take part in the study. Participants were included if they: (a) were 18 years or above, (b) had personally experienced the 6 February 2023 earthquake, (c) reported elevated levels of psychological distress (indicated by a score of >15 on the Kessler-10 Psychological Distress Scale (K10)^29^ and (d) had reduced psychosocial functioning (indicated by a score of >16 on the World Health Organization Disability Assessment Schedule (WHODAS 2.0).^30^ These cut-off scores were determined in line with screening procedures recommended in the PM+ manual^20^ and previous PM+ trials^25,26^. The Turkish versions of these instruments have demonstrated good reliability and validity^31,32^ (see Outcome Measures section). The exclusion criteria were (a) acute medical conditions, (b) imminent suicide risk (evaluated using the suicidal thoughts interview of the PM+ manual), (c) severe mental disorders (e.g. psychotic disorders or substance use disorder), (d) cognitive impairment (e.g. severe intellectual disability, assessed using the PM+ manual observation checklist) or (e) ongoing treatment in specialised mental health care. Participants excluded due to acute suicide risk (*n* = 4) were referred to local psychiatric units for specialised care; no additional referrals were required for other exclusion reasons (Fig. 1).

Recruitment, screening and baseline assessments were conducted in three waves between 17 November 2023, and 10 June 2024, approximately 9 months after the 6 February 2023 earthquakes, by trained DEPSDA staff. All study procedures, including intervention sessions, post- and follow-up assessments, were completed on 30 August 2024. Post-assessments were conducted 1 week after and follow-up assessments were conducted 1 month after the final session of PM+. After completion of the follow-up assessment of the intervention group, those in the control condition were offered PM+.

### Procedure

#### Randomisation and blinding

Eligible participants were randomised 1:1 to PM+ or E-CAU. A randomisation list with random block sizes 4 and 6 was generated in Stata version 17 for macOS (StataCorp LLC, College Station, Texas, USA; https://www.stata.com) through the ‘ralloc’ command^33^ by an independent researcher not involved in the rest of the study. The personnel responsible for enrolment and intervention delivery had no access to the allocation sequence. Participants were not blind to their study condition because of the nature of the intervention. Outcome assessors were blinded; they did not have access to the randomisation list and were not involved in the intervention delivery.

### Intervention

PM+ intervention consists of five weekly in-person sessions lasting 90 min. It integrates four evidence-based behavioural strategies: (a) stress management using slow breathing to manage anxiety and stress reactions (session 1), (b) a structured problem-management approach to help participants address practical problems such as unemployment or relational conflict (session 2), (c) behavioural activation through gradual re-engagement with pleasant and task-oriented activities to enhance mood and functionality (session 3) and (d) strengthening social support by guiding participants to identify supportive individuals and seek emotional or practical support from their social networks (session 4). The first session also includes psychoeducation about common psychological reactions to adversity and the rationale of the intervention strategies. In each session, homework practice is assigned and reviewed in the following session. The fifth session focuses on reviewing all strategies and discussing relapse prevention options. The facilitator training of the PM+ was adapted to the earthquake context for this study. Case studies within the existing training manual were modified to reflect scenarios relevant to earthquake survivors that might be encountered in the field. The training consisted of a 6-day online course conducted by one master PM+ trainer and supervisor, and one PM+ trainer (A.C.T and C.A.). The training covered basic helping skills, PM+ delivery, assessment protocols and suicide risk management.

PM+ was delivered individually in face-to-face sessions at DEPSDA offices located within container cities by eight early-career psychologists from DEPSDA, working across four cities. The PM+ facilitators (one male and seven female) were psychology graduates with 6 to 10 months of experience providing psychosocial support in the earthquake-affected field. During the implementation of the intervention, facilitators received weekly online group supervision by the trainers. The supervisions aimed to monitor the intervention delivery process to ensure fidelity and to support the facilitators when needed, including monitoring any harms. Participants were routinely monitored for changes in risk, including distress and suicidality, through structured check-ins at the beginning of each session, and any concerns were reviewed in supervision. No additional participants were identified as requiring referral for psychiatric assessment. Treatment completion was defined as attending all five sessions.

### E-CAU

E-CAU referred to usual care that was enhanced by providing the control group with clear information about any available MHPSS services offered by public institutions and NGOs operating in the container cities. These services typically included brief psychological support, psychosocial group activities (e.g. psychoeducation, recreational or community-based activities) or referral pathways to specialised care (e.g. psychiatry units or psychotherapy services), depending on local availability. Participants were also given guidance on how to access these services. Uptake of additional mental health services among E-CAU participants was recorded at each assessment point. Participants were also monitored at assessment time points for signs of elevated risk, and no need for psychiatric referral was identified.

### Outcome measures

The primary outcome was the symptom levels of depression, assessed using the Patient Health Questionnaire-9 (PHQ-9).^34^ The PHQ-9 is a nine-item measure assessing depressive symptoms over the last 2 weeks. Each item is rated on a four-point scale, with total scores ranging from 0 to 27. Higher scores indicate more severe symptoms. The PHQ-9 has demonstrated good psychometric properties across several resource-constrained settings^35^ and has been adapted into Turkish with a Cronbach’s α of 0.84.^36^

Secondary outcomes included self-identified problems, functional impairment, psychological distress, anxiety and PTSD. Self-identified problems were measured with the Psychological Outcome Profiles (PSYCHLOPS),^37^ which assesses two main problems, functioning and well-being. Each response is rated on a six-point scale, with a maximum total score of 20, where higher scores indicate greater severity. The scale has been validated in previous research.^38,39^ Functional impairment was assessed using WHODAS 2.0,^30^ a 12-item measure of daily functioning over the past 30 days. Each item is scored on a five-point Likert scale, yielding a total score between 12 and 60, with higher scores indicating more dysfunction. WHODAS has good psychometric properties across many countries^40^ and has been adapted to Turkish with good validity and reliability.^31^ Psychological distress was measured using the K-10),^29^ a ten-item questionnaire designed to measure generalised or non-specific psychological stress. Each item is rated on a five-point scale, resulting in a total score between 10 and 50, where higher scores reflect more severe distress. K10 has shown good psychometric properties across various populations,^41,42^ and its Turkish adaptation has shown good reliability with a Cronbach’s α of 0.94.^32^ Anxiety symptoms were assessed using the Generalised Anxiety Disorder Scale (GAD-7),^43^ a seven-item measure of general anxiety symptoms over the last 2 weeks. Each item is rated on a four-point scale, with a total score between 0 and 21, where higher scores indicate greater anxiety. The GAD-7 is a reliable and valid measure in both clinical^44^ and general populations,^45^ with a Turkish adaptation showing good psychometric properties (Cronbach’s α = 0.85).^46^ PTSD symptoms were measured using the Post-traumatic Stress Disorder Checklist for DSM-5 (PCL-5),^47^ a 20-item measure rated on a 5-point scale, yielding a total score between 0 and 80, where higher scores indicate more severe PTSD. PCL-5 has demonstrated strong psychometric properties^48^ and has been adapted to Turkish with good reliability and validity (Cronbach’s α = 0.94).^49^ Internal consistency in the current sample was acceptable to excellent across the outcome measures (Cronbach’s α = 0.85 for PHQ-9, 0.68 for PSYCHLOPS, 0.86 for WHODAS 2.0, 0.92 for K10, 0.87 for GAD-7 and 0.93 for PCL-5).

Information on key demographic characteristics such as gender, age, marital status, education and occupational status were collected. All assessments were conducted in person by trained DEPSDA staff who were blind to the group allocation of the participants, at baseline, 1 week post intervention (a week after the last session of the intervention) and the 1-month follow-up (1 month after the last session of the intervention). Since some participants had limited literacy, assessors verbally administered the questions and recorded the participants’ responses on hard-copy scales.

### Statistical analysis

All analyses were conducted according to the intention-to-treat (ITT) principle in R studio version 4.5.3 for macOS (R Foundation for Statistical Computing, Vienna, Austria; https://www.r-project.org). We tested the effect of each treatment condition on primary outcome, depression (PHQ-9) and secondary outcomes: self-identified problems (PSYCHLOPS), functional impairment (WHODAS 2.0), psychological distress (K-10), anxiety (GAD-7) and PTSD symptoms (PCL-5).

Linear mixed models (LMMs) were used to estimate treatment effects at each time point, comparing the changes from baseline to each assessment. Models were estimated using maximum likelihood estimation, which uses all available data and makes valid inferences under the missing-at-random assumption. To estimate the overall treatment effect across assessment points, two dummy variables for time were created: one for the 1-week post-assessment and another for the 1-month follow-up assessment, with baseline as the reference category. Interaction terms between the time variables and condition were then created to examine whether changes observed in the intervention group significantly differed from those in the control group. The coefficients of the interaction terms refer to the adjusted mean differences between the two conditions at each point, thereby providing the estimates of treatment effects. All models included a random intercept for participants to allow individual-specific baseline scores on outcomes. As the condition itself was not included in the models, the intercept represents the average baseline score of all the participants across both conditions. The analyses were repeated by adding gender and age as covariates. To assess the robustness of the results, a subsequent analysis was also conducted, including only participants who completed the 1-week post-assessment (completers-only analysis).

## Results

### Participants

A total of 90 participants were screened, of whom 76 were found to be eligible and randomly assigned to PM+ (*n* = 38) and E-CAU (*n* = 38). Figure 1 presents the Consort flow diagram. Of the included participants, 64 were female (84.2%). The mean age of participants was 33.36 (s.d. = 10.99). The majority were married (36, 47.4%) and homemakers (29, 38.2%). Baseline characteristics were not significantly different between the two groups. The sample characteristics are presented in Table 1.

**Table 1:** Baseline sample characteristics

Characteristics	Total (*N* = 76)	PM+ (*N* = 38)	E-CAU (*N* = 38)	*t*/χ^2^	*P*-value
Female, *n* (%)	64 (84.2)	34 (89.5)	30 (78.9)	1.58	0.208
Age, years, mean (s.d.)	33.36 (10.99)	32.53 (11.68)	34.25 (10.34)	−0.63	0.530
Marital status, *n* (%)				2.799	0.633
Never married	24 (31.6)	14 (36.8)	10 (26.3)		
Married	42 (55.3)	19 (50.0)	23 (60.5)		
Separated	3 (3.9)	1 (2.6)	2 (5.3)		
Divorced	4 (5.3)	3 (7.9)	1 (2.6)		
Widowed	3 (3.9)	1 (2.6)	2 (5.3)		
Education, *n* (%)				1.308	0.860
None	3 (3.9)	2 (5.3)	1 (2.6)		
Primary school or less	15 (19.7)	7 (18.4)	8 (21.1)		
Middle school	10 (13.2)	4 (10.5)	6 (15.8)		
High school	29 (38.2)	14 (36.8)	15 (39.5)		
University	19 (25.0)	11 (28.9)	8 (21.1)		
Occupation status, *n* (%)				3.906	0.774
Salaried job	12 (15.8)	6 (15.8)	6 (15.8)		
Self-employed	6 (7.9)	2 (5.3)	4 (10.5)		
Unpaid volunteer work	2 (2.6)	2 (5.3)	0 (0)		
Student	14 (18.4)	8 (21.1)	6 (15.8)		
Homemaker	35 (46.1)	16 (42.1)	19 (50.0)		
Retired	1 (1.3)	1 (2.6)	0 (0)		
Unemployed	6 (7.9)	3 (7.9)	3 (7.9)		
Field, *n* (%)				0.982	0.806
Adıyaman	19 (25.0)	9 (23.7)	10 (26.3)		
Kahramanmaraş	17 (22.4)	7 (18.4)	10 (26.3)		
Malatya	20 (26.3)	11 (28.9)	9 (23.7)		
Osmaniye	20 (26.3)	11 (28.9)	9 (23.7)		

PM+, Problem Management Plus, E-CAU, enhanced care-as-usual.

The primary outcome measure (1-week post-assessment) was completed for 56 participants (*n* = 30 in PM+ and *n* = 26 in E-CAU). Participants who were lost to post-assessment did not differ significantly from those who were retained on any demographic or baseline characteristics (Table 2).

**Table 2:** Baseline sample characteristics of retained at and lost to post-test participants

Characteristics	Retained at post-test (*n* = 56)	Lost to post-test (*n* = 20)	*t*/χ^2^	*P*-value
Female, *n* (%)	47 (83.9)	17 (85)	0.013	0.910
Age, years, mean (s.d.)	33.82 (11.51)	30.80 (7.45)	0.798	0.428
Marital status, *n* (%)			2.971	0.563
Never married	19 (33.9)	5 (25.0)		
Married	31 (55.4)	11 (55.0)		
Separated	2 (3.6)	1 (5.0)		
Divorced	3 (5.4)	1 (5.0)		
Widowed	1 (1.8)	2 (10.0)		
Education, *n* (%)			4.915	0.296
None	2 (3.6)	1 (5.0)		
Primary school or less	12 (21.4)	3 (15.0)		
Middle school	7 (12.5)	3 (15.0)		
High school	18 (32.1)	11 (55.0)		
University	17 (30.4)	2 (10.0)		
Occupation status, *n* (%)			5.605	0.469
Salaried job	11 (19.6)	1 (5.0)		
Self-employed	4 (7.1)	2 (10.0)		
Unpaid volunteer work	2 (3.6)	0 (0)		
Student	9 (16.1)	5 (25.0)		
Homemaker	26 (46.4)	9 (45.0)		
Retired	1 (1.8)	0 (0)		
Unemployed	3 (5.4)	3 (15.0)		
Field, *n* (%)			2.408	0.492
Adıyaman	13 (23.2)	4 (20)		
Kahramanmaraş	15 (26.8)	2 (20)		
Malatya	14 (25.0)	6 (20)		
Osmaniye	14 (25.0)	6 (20)		

### Feasibility outcomes

Retention from baseline to post-assessment was 74% (56 of 76 participants), corresponding to an attrition rate of 26%. Data completeness was high: all 6 multi-item measures were fully completed at baseline, and at post-assessment only 1 measure was missing for 4 of the 56 participants. Of the 38 participants randomised to the PM+ condition, 30 (79%) completed the treatment, all of whom attended every session (Fig. 1). Of the eight participants who dropped out, five did not attend any sessions, one discontinued after the first session, and two discontinued after two sessions. Common reasons for not attending sessions were sickness, caregiving responsibilities, unstable housing conditions and relocation. No serious adverse events were reported during the study.

The study was conducted in a challenging post-disaster context with ongoing stressors such as limited access to water and electricity, frequent aftershocks and occasional emergencies such as fires and flooding. Sometimes these disruptions prevented regular session attendance and required flexible adjustments to schedules. Attendance to follow-up assessments was lower, particularly in the control group. Field notes documented high mobility of survivors and multiple concurrent surveys conducted by various organisations in the region as factors affecting follow-up assessment completion.

Fidelity to the intervention protocol was checked through weekly group supervision. Although no formal fidelity checklist was used, supervisors monitored the delivery process to ensure adherence to the intervention protocol. Supervisions also focused on addressing contextual barriers to support facilitators in adapting to changing field conditions and facilitator’s own self-care. In supervision, participants’ presenting problems were most frequently reported as relational conflicts within families, as well as economic hardship, unemployment and uncertainty about the future, alongside distress related to aftershocks, insomnia, anger and social withdrawal.

### Effectiveness of PM+ on primary and secondary outcomes

ITT analysis showed a significant difference between the PM+ and E-CAU in primary outcome (PHQ-9) at the 1-week post-assessment. Participants in the PM+ condition reported a greater improvement in depression symptoms than those in the E-CAU condition (adjusted mean difference −5.91, 95% CI −8.23, −3.58, *p* = 0.000, *d* = 1.09). Similarly, participants in the PM+ condition demonstrated significantly greater reductions than those in the E-CAU condition on all secondary outcome measures at the 1-week post-assessment (adjusted mean difference −6.94, 95% CI −8.88, −5.01, *p* = 0.000, *d* = 1.62 for PSYCHLOPS; adjusted mean difference −8.77, 95% CI −12.50, −5.03, *p* = 0.000, *d* = 1.05 for WHODAS; adjusted mean difference −9.66, 95% CI −13.35, −5.97, *p* = 0.000, *d* = 1.17 for K-10; adjusted mean difference −4.31, 95% CI −6.39, −2.24, *p* = 0.000, *d* = 0.93 for GAD-7; and adjusted mean difference −18.72, 95% CI −25.97, −11.47, *p* = 0.000, *d* = 1.10 for PCL-5).

At the 1-month follow-up assessment, significant differences between PM+ and E-CAU remained significant only for PSYCHLOPS (adjusted mean difference −5.96, 95% CI −9.44, −2.45, *p* = 0.001, *d* = 1.13), and K-10 (adjusted mean difference −6.78, 95% CI −12.73, −0.79, *p* = 0.029, *d* = 0.76), with participants in the PM+ condition having greater reductions than those in E-CAU. However, no significant differences were observed for the primary outcome (PHQ-9) and other secondary outcome measures at this time point.

Planned covariate-adjusted analyses (controlled for gender and age) yielded results largely consistent with the primary analyses. After adjusting for covariates, in addition to the PSYCHLOPS and K-10 at follow-up, significant differences between PM+ and E-CAU were also observed for changes in WHODAS (adjusted mean difference −6.69, 95% CI −12.43, −0.84, *p* = 0.027, *d* = 1.08), and PCL-5 (adjusted mean difference −12.36, 95% CI −23.99, −0.27, *p* = 0.042, *d* = 0.76), indicating greater reductions in scores for the PM+ condition. Adjusting for covariates did not eliminate any of the significant effects observed at either time point. All ITT results are presented in Table 3.

**Table 3:** Results from mixed-model analysis of primary and secondary outcomes for ITT sample

Outcome variable	Time point	Descriptive statistics		Mixed-model analysis			Covariate-adjusted mixed model analysis		
		Mean (s.d.)	Mean (s.d.)						
		PM+ (*n* = 38)	E-CAU (*n* = 38)	Difference in mean	*P*-value	Effect size	Difference in mean	*P*-value	Effect size
PHQ-9	Baseline	14.55 (5.04)	13.13 (7.20)						
	Post	6.37 (3.84)	11.12 (6.64)	−5.91 (−8.23, −3.58)	0.000	1.09	−6.12 (−8.41, −3.81)	0.000	1.13
	1 month	8.94 (5.77)	8.71 (3.77)	−3.24 (−6.97, −0.51)	0.093	0.66	−3.70 (−7.39, 0.04)	0.056	0.76
PSYCHLOPS	Baseline	15.66 (2.52)	14.24 (4.47)						
	Post	5.97 (4.19)	12.46 (4.40)	−6.94 (−8.88, −5.01)	0.000	1.62	−6.90 (−8.85, −4.92)	0.000	1.61
	1 month	7.80 (4.61)	11.86 (5.90)	−5.96 (−9.44, −2.45)	0.001	1.13	−6.15 (−9.62, −2.57)	0.001	1.16
WHODAS	Baseline	31.42 (9.11)	28.92 (10.43)						
	Post	20.50 (5.42)	27.04 (10.51)	−8.77 (−12.50, −5.03)	0.000	1.05	−9.04 (−12.71, −5.31)	0.000	1.08
	1 month	20.56 (6.50)	20.43 (5.88)	−5.81 (−11.62, 0.04)	0.053	0.94	−6.69 (−12.43, −0.84)	0.027	1.08
K-10	Baseline	31.53 (8.40)	30.79 (10.71)						
	Post	18.03 (6.76)	27.88 (9.51)	−9.66 (−13.35, −5.97)	0.000	1.17	−10.03 (−13.72, −6.38)	0.000	1.22
	1 month	21.06 (7.43)	23.86 (10.22)	−6.78 (−12.73, −0.79)	0.029	0.76	−7.76 (−13.68, −1.80)	0.013	0.87
GAD-7	Baseline	12.08 (4.36)	11.61 (6.05)						
	Post	5.63 (3.51)	9.81 (5.53)	−4.31 (−6.39, −2.24)	0.000	0.93	−4.42 (−6.47, −2.38)	0.000	0.96
	1 month	7.06 (4.33)	7.29 (3.30)	−2.30 (−5.67, 1.08)	0.186	0.60	−2.71 (−6.03, 0.63)	0.118	0.71
PCL-5	Baseline	49.82 (13.20)	44.21 (20.84)						
	Post	24.20 (13.62)	39.00 (19.95)	−18.72 (−25.97, −11.47)	0.000	1.10	−19.33 (−26.48, −12.06)	0.000	1.13
	1 month	28.19 (17.64)	21.86 (14.74)	−10.75 (−22.62, 1.27)	0.075	0.66	−12.36 (−23.99, −0.28)	0.042	0.76

ITT, intention-to-treat; PM+, Problem Management Plus; E-CAU, enhanced care-as-usual; PHQ-9, Patient Health Questionnaire; PSYCHLOPS, Psychological Outcome Profiles; WHODAS, World Health Organization Disability Assessment Schedule; K-10, Kessler-10 Psychological Distress Scale; GAD-7, Generalised Anxiety Disorder Scale; PCL-5, Post-traumatic Stress Disorder Checklist.

The completers-only analysis, focusing on participants who were retained at the 1-week post-assessment, yielded the same results as the primary ITT analyses (Table 4). Results from the covariate-adjusted analysis in the completers-only sample were also consistent with those of the covariate-adjusted ITT analysis. Further significant differences emerged for the changes in WHODAS (adjusted mean difference −6.67, 95% CI −12.34, −0.87, *p* = 0.026, *d* = 1.08) and PCL-5 (adjusted mean difference −12.33, 95% CI −23.73, −0.45, *p* = 0.039, *d* = 0.76) scores, with greater reductions in the PM+ condition compared with E-CAU. All completers-only analysis results are presented in Table 4.

**Table 4:** Results from mixed-model analysis of primary and secondary outcomes for completers-only sample Table 4 long description.

Outcome variable	Time point	Descriptive statistics		Mixed-model analysis			Covariate-adjusted mixed model analysis		
		Mean (s.d.)	Mean (s.d.)						
		PM+ (*n* = 30)	E-CAU (*n* = 26)	Difference in mean	*P*-value	Effect size	Difference in mean	*P*-value	Effect size
PHQ-9	Baseline	14.80 (5.18)	13.04 (6.59)						
	Post	6.37 (3.84)	11.12 (6.64)	−5.85 (−8.12, −3.57)	0.000	1.08	−6.11 (−8.36, −3.82)	0.000	1.13
	1 month	8.94 (5.77)	8.71 (3.77)	−3.11 (−6.78, −0.58)	0.101	0.64	−3.62 (−7.25, 0.09)	0.059	0.74
PSYCHLOPS	Baseline	15.83 (2.29)	14.77 (4.29)						
	Post	5.97 (4.19)	12.46 (4.40)	−6.93 (−8.85, −5.02)	0.000	1.61	−6.97 (−8.89, −5.03)	0.000	1.62
	1 month	7.80 (4.61)	11.86 (5.90)	−5.93 (−9.38, −2.45)	0.001	1.12	−6.21 (−9.63, −2.67)	0.000	1.17
WHODAS	Baseline	32.17 (9.78)	29.50 (9.71)						
	Post	20.50 (5.42)	27.04 (10.51)	−8.72 (−12.41, −5.01)	0.000	1.04	−9.05 (−12.68, −5.37)	0.000	1.08
	1 month	20.56 (6.50)	20.43 (5.88)	−5.71 (−11.47, 0.11)	0.056	0.92	−6.67 (−12.34, −0.87)	0.026	1.08
K-10	Baseline	31.03 (8.52)	31.35 (9.84)						
	Post	18.03 (6.76)	27.88 (9.51)	−9.67 (−13.28, −6.06)	0.000	1.17	−10.09 (−13.71, −6.51)	0.000	1.22
	1 month	21.06 (7.43)	23.86 (10.22)	−6.65 (−12.49, −0.77)	0.029	0.74	−7.70 (−13.53, −1.83)	0.012	0.86
GAD-7	Baseline	11.97 (4.50)	11.73 (5.59)						
	Post	5.63 (3.51)	9.81 (5.53)	−4.30 (−6.33, −2.28)	0.000	0.93	−4.48 (−6.51, −2.46)	0.000	0.97
	1 month	7.06 (4.33)	7.29 (3.30)	−2.22 (−5.52, 1.09)	0.193	0.58	−2.74 (−6.03, 0.58)	0.112	0.71
PCL-5	Baseline	50.17 (13.64)	43.77 (19.02)						
	Post	24.20 (13.62)	39.00 (19.95)	−18.56 (−25.70, −11.42)	0.000	1.09	−19.47 (−26.46, −12.38)	0.000	1.14
	1 month	28.19 (17.64)	21.86 (14.74)	−10.26 (−22.01, 1.67)	0.085	0.63	−12.33 (−23.73, −0.45)	0.039	0.76

PM+, Problem Management Plus; E-CAU, enhanced care-as-usual; PHQ-9, Patient Health Questionnaire; PSYCHLOPS, Psychological Outcome Profiles; WHODAS, World Health Organization Disability Assessment Schedule; K-10, Kessler-10 Psychological Distress Scale; GAD-7, Generalised Anxiety Disorder Scale; PCL-5, Post-traumatic Stress Disorder Checklist.

As eight participants in the E-CAU group reported receiving other mental health services at post-assessment (cognitive behavioural therapy [CBT; *n* = 4], eye movement desensitisation and reprocessing [EMDR; *n* = 2] and psychosocial support [*n* = 2]) provided by local organisations, we conducted an exploratory ITT analysis excluding these participants. Three of them (two receiving CBT and one EMDR) continued treatment during the follow-up period, whereas the other five were lost to follow-up. The results remained consistent with the primary ITT findings; however, at follow-up, further significant differences emerged for changes in WHODAS (adjusted mean difference −7.36, 95% CI −14.38, −0.28, *p* = 0.045, *d* = 1.02), GAD-7 (adjusted mean difference −5.99, 95% CI −9.69, −2.27, *p* = 0.002, *d* = 1.86) and PCL-5 (adjusted mean difference −17.85, 95% CI −31.79, −3.67, *p* = 0.013, *d* = 1.02) with greater reductions in the PM+ group compared with E-CAU (Supplementary Material 2, Table S2).

## Discussion

This pilot RCT provides preliminary evidence that PM+ is a feasible and potentially effective intervention for reducing depression and other common mental health problems among Turkish earthquake survivors. Participants in the PM+ condition showed greater improvements than those receiving E-CAU across all primary and secondary outcomes at post-treatment, with partial maintenance of effects at 1-month follow-up. Recruitment, retention and adherence outcomes supported the feasibility and acceptability of PM+ in the post-disaster context.

Implementation and recruitment were facilitated by collaborating with an NGO working with earthquake survivors. Participants who began the sessions showed strong engagement, as nearly all completers attended every session, indicating good adherence. Attrition only occurred before or during the first sessions, often due to displacement, caregiving responsibilities or health problems – typical barriers in crisis-affected populations. Retention to post-assessment (74%) and treatment adherence (79% completion) were comparable to previous PM+ trials in humanitarian settings.^23,24,26^ The only other PM+ study conducted among earthquake-affected communities is a feasibility trial of gPM+ in Nepal, which reported higher retention (97.5%) and strong session attendance.^50^ However, compared with that study, which was conducted in a relatively more stable rural setting, the present findings reflect feasibility under ongoing post-disaster stressors and continued instability. The low follow-up rate underscores the challenges in maintaining engagement in highly mobile post-disaster environments. Similar to other studies, the transitory and unstable living conditions of survivors have been shown to hamper longer-term participation, leading to substantial loss to follow-up despite initial engagement.^51,52^ In addition, follow-up engagement may also have been affected by survey fatigue from several concurrent assessments in the field. These findings suggest that feasibility may be limited by contextual and disaster-specific barriers rather than intervention acceptability. Strategies such as flexible session scheduling to adapt to field conditions or childcare support could help reduce attrition and enhance accessibility in future trials.^53^

Participants receiving PM+ reported significantly greater improvements in depression, psychological distress, anxiety, PTSD symptoms, functional impairment and self-identified problems at post-treatment compared with those receiving E-CAU. The treatment effects were partially maintained at 1-month follow-up, with significant group differences only reported in self-identified problems and psychological distress. These findings are mainly consistent with previous PM+ trials conducted in humanitarian settings, which have reported improvements in depression, psychological distress and self-identified problems, while findings for PTSD, anxiety and functional impairment have been more heterogeneous.^21–24,54^ Most previous PM+ trials have been conducted with conflict-affected or refugee populations in both low and high resource settings,^21–24,54^ and evidence from earthquake-affected populations remains scarce, with only a non-powered feasibility trial in Nepal.^50^ Both studies reported improvements across outcomes; however, the present findings extend this evidence by demonstrating effects in an individually delivered format by early-career psychologists and by providing preliminary evidence among earthquake survivors living in temporary post-disaster settlements. The largest effect size was consistently observed for self-identified problems in all analyses. In this sample, relational conflicts were the most frequently reported self-identified problems, consistent with evidence highlighting relational difficulties as among the most salient sources of distress in post-disaster contexts.^55^ Supervision notes indicated these interpersonal problems often involved marital conflict, parenting challenges and tensions with in-laws, exacerbated by the lack of privacy and overcrowded conditions in container homes. Participants frequently reported that PM+ strategies, especially problem management, helped them cope with these relational stressors. This suggests that the intervention may be particularly helpful in addressing interpersonal problems, and highlights its potential for settings where disrupted post-disaster living conditions heighten relational tensions.

PM+ led to significant improvements across all outcomes at post-treatment; however, effects were partially maintained at the follow-up, with significant group differences remaining only in self-identified problems and psychological distress. This pattern of decline over time is consistent with previous PM+ trials ^24,25^ and may reflect the challenges of maintaining gains from low-intensity interventions in unstable and adverse settings. One explanation may lie in the ongoing stressors such as joblessness, economic hardship, limited access to resources, prolonged water and electricity shortages, and frequent aftershocks reactivating trauma symptoms even a year after. Moreover, some E-CAU participants accessed other mental health services during the trial, which may have diluted between-group differences at follow-up. Exploratory analyses excluding these participants showed additional significant between-group differences for functional impairment, anxiety and PTSD at follow-up, suggesting that concurrent treatment use likely attenuated the effects rather than indicating the limited effectiveness of PM+. Overall, these findings may support the robustness of PM+ in this post-disaster context, despite additional mental health service use among some control participants. Finally, the absence of booster sessions may have contributed to the partial loss of gains over time. These findings emphasise the critical role of exploring augmentation strategies, such as booster sessions or stepped-care approaches, to help maintain the benefits of low-intensity interventions in complex post-disaster settings.^25^

### Implementation context

Although PM+ was designed for delivery by trained non-professionals, in this study it was implemented by early-career psychologists, since national regulations permitted only MHPSS workers to deliver psychological interventions in disaster areas. In Türkiye, mental health care faces challenges such as shortage of specialised professionals as well as service quality and limited standardised structures across existing services.^18^ Following the earthquakes, many MHPSS providers were mobilised, but their competencies and organisational and supervisory support structures varied considerably. Within this context, PM+ offered not only a brief, evidence-based intervention but also a structured framework and standardised training and supervision, which might strengthen the consistency and quality of service delivery. Supervision notes indicated that this structure provided clear guidance and enhanced facilitators’ confidence and effectiveness in navigating chaotic, resource-limited field conditions. Within the DEPSDA initiative, PM+ provided preliminary insights into how such a model can strengthen the competencies of early-career field workers and service delivery in post-disaster settings. These findings suggest the potential of PM+ as a capacity-building model and a scalable tool that could contribute to the Ministry of Health’s ongoing efforts to improve community-based mental health services, as outlined in the National Mental Health Action Plan.^56^ PM+ may also be suitable for integration into national post-disaster psychosocial response systems. While delivery in this study was limited to MHPSS professionals, PM+ was originally designed for trained non-specialists and can be implemented through community-based providers (e.g. health workers, teachers or clergy) to enhance reach, acceptability and scalability where such delivery models are feasible. By evaluating PM+ in the early post-disaster recovery period, when displacement, infrastructure disruption and service instability were still prominent, this pilot study provides preliminary, context-specific insights into its delivery in a challenging and under-represented setting, contributing to the implementation evidence gaps highlighted in recent reviews.^57^ Therefore, this pilot study offers early evidence that can inform future efforts to reduce the know–do gap in post-disaster mental healthcare.^58^

### Strengths, limitations and future directions

This study has several strengths including the use of a randomised controlled design with an active comparison group and multiple post-treatment assessment points, and its evaluation of a scalable psychological intervention among earthquake survivors in a real-world setting following a large-scale disaster, providing feasibility insights to inform future fully powered trials. The study benefited from regular supervision and recording of any psychological treatments received by E-CAU participants, which allows transparency regarding additional mental health service use during the trial.^28^ The study also has several limitations. Given the pilot nature of the present study, our sample size was small, which limited statistical power to draw stronger conclusions about the effectiveness of PM+. Another limitation of the study was the high follow-up attrition rate (39.5%), similar to other humanitarian trials.^21^ Exploratory analyses revealed no demographic differences between retained and drop-out participants. The loss to follow-up might be explained by field conditions such as relocations, survey fatigue and logistical challenges in tracking participants. The outcomes were assessed through self-report measures, which may have led to response bias. Fidelity was monitored through weekly supervision; however, the absence of a formal fidelity checklist limited objective verification of protocol adherence. The 1-month follow-up period limited the ability to explore whether the intervention gains were sustainable over time. Future studies should include longer follow-up periods and investigate the effects of booster sessions to maintain treatment gains. Future research may also examine the feasibility of family-based interventions, particularly in addressing relational distress in post-disaster settings. The study did not include measures of earthquake exposure (e.g. injury or bereavement), which may have contributed to variability in psychological outcomes. The study did not assess participants’ chronic illnesses or medication use, which might have influenced psychological outcomes and functional impairment scores. Similarly, given the widespread experience of multiple losses in this population, co-occurring grief may have overlapped with symptoms captured by the study measures. Future research should incorporate assessments of earthquake exposure, physical health conditions and medication use, and grief-specific measures. Overall, findings provided preliminary evidence that PM+ is both feasible and potentially effective in a setting where chronic stressors and unstable service structures complicate psychosocial care. A fully powered definitive trial is needed to test its long-term effectiveness and whether it can be sustainably integrated into Türkiye’s mental health response system.

## Supporting information

10.1192/bjo.2026.12016.sm001 Coşkun Toker et al. supplementary material Coşkun Toker et al. supplementary material

## Data Availability

The anonymised data that support the findings of this study are available from the corresponding author (C.A.) upon reasonable request. The data are not publicly available due to ethical restrictions and the sensitive nature of post-disaster mental health information.

## Supplementary material

The supplementary material is available online at https://doi.org/10.1192/bjo.2026.12016

## Fig. 1 Long description

The flowchart details the process of a clinical trial. It begins with enrolment where 90 participants are assessed for eligibility. 14 participants are excluded for various reasons such as not meeting inclusion criteria, WHODAS scores, K-10 scores, thoughts of suicide, and cognitive impairment. 76 participants are randomised into two groups: intervention and E-CAU. In the intervention group, 38 participants are allocated, with 30 receiving the intervention and 8 not receiving it due to reasons like sickness and responsibilities. In the E-CAU group, 38 participants are allocated and all receive the care. Post-assessment shows no loss in the intervention group but 12 participants are lost in the E-CAU group. Follow-up at 3 weeks shows 14 participants lost in the intervention group and 19 in the E-CAU group. Finally, 38 participants from each group are analysed.

Navigate back to Fig. 1.

## Table 4 Long description

The table presents data on descriptive statistics, mixed-model analysis, and covariate-adjusted mixed model analysis for various outcome variables at different time points. It includes columns for mean and standard deviation (s.d.) at baseline, post, and 1 month for two groups (PM+ and E-CAU), as well as columns for the difference in mean, P-value, and effect size for both mixed-model analysis and covariate-adjusted mixed model analysis. The outcome variables include PHQ-9, PSYCLOPS, WHODAS, K-10, GAD-7, and PCL-5. Each row provides data for a specific outcome variable and time point. Notable trends include significant differences in mean values for various outcome variables at different time points, with corresponding P-values and effect sizes indicating the statistical significance and magnitude of these differences.

Navigate back to Table 4.
